# Virtual reality visualization of patient specific heart model

**DOI:** 10.1186/1532-429X-18-S1-T13

**Published:** 2016-01-27

**Authors:** Matthew Bramlet, Kuocheng Wang, Alexander Clemons, Nathaniel Christopher Speidel, Steven M Lavalle, Thenkurussi Kesavadas

**Affiliations:** 1 grid.35403.310000000419369991Health Care Engineering Systems, University of Illinois at Urbana-Champaign, Urbana, IL USA; 2 grid.430852.80000000107414132Pediatric Cardiology, Children's Hospital of Illinois, University of Illinois College of Medicine at Peoria, Peoria, IL USA; 3 grid.35403.310000000419369991Computer Science, University of Illinois at Urbana-Champaign, Urbana, IL USA

## Background

The diagnostic dilemma that arises with congenital cardiac anomalies is visualizing the 3-D complexity of a child's heart with 2-D tools. The complicated intracardiac relationships and extracardiac connections make it challenging to diagnose the defect and plan treatment for the child. It has been demonstrated that it is difficult to reconstruct an accurate mental 3D model from 2D images [Guillot 2007], yet we rely on this imperfect modality to make critical surgical decisions. 3D printed models are proving to be highly valuable in improving the understanding of these complex defects, but require many resources. In this project we have explored 3D immersive virtual reality technology to provide deeper understanding and visualization of cardiac models and supplant the need for a physical printed model. Using stereoscopic 3D in the form of 3D displays on head mounted displays (HMDs) excellent intuitive model was created. Models were generated from patient specific MRI which was converted to format that was compatible with virtual reality rendering.

## Methods

The source images were obtained from a 3D respiratory-navigated balanced SSFP (steady state free precession) multi-slab sequence with T2 preparation. The 3D data set was imported into a segmentation software (Mimics) where a mask was applied over the myocardium and vessel walls and converted into a stereolithography file (STL) which was then converted to either filmbox format (FBX) or 3DS format using Blender. The FBX file is loaded in a 3D environment supported by the Oculus Development Kit 2 (DK2) HMD. In this case, the Unreal 4 engine is used to show and manipulate the FBX file. Three manipulation input devices including a keyboard and mouse, the Nintendo Wii Remote and the Microsoft Kinect were tested. Further an Augmented Reality image based tracking system was also tested.

## Results

The Virtual Reality based visualization of the heart was implemented successfully. The system provided intuitive control to allow the user to manipulate the heart into relatable positions allowing for diagnostic assessment of the complex congenital defects. In terms of implementation several challenges were faced. With the Unreal 4 engine, gimbal lock posed an issue controlling visualization with three degrees of freedom in rotation. To provide a temporary fix to this, only two degrees, yaw and roll, were allowed. The keyboard and mouse controls were preferred over other forms of manipulation in the Unreal 4 engine. The Wii Remote posed problems because, while the tracking using the sensor bar is fairly accurate, the data from the Wii Motion Plus is very difficult to access and acceleration data led to drift error.

## Conclusions

The virtual reality based visualization of the heart model shows a great deal of promise to deepen understanding of a patient specific anatomy and has the potential of becoming an important diagnostic asset in the future.

**Figure 1:**
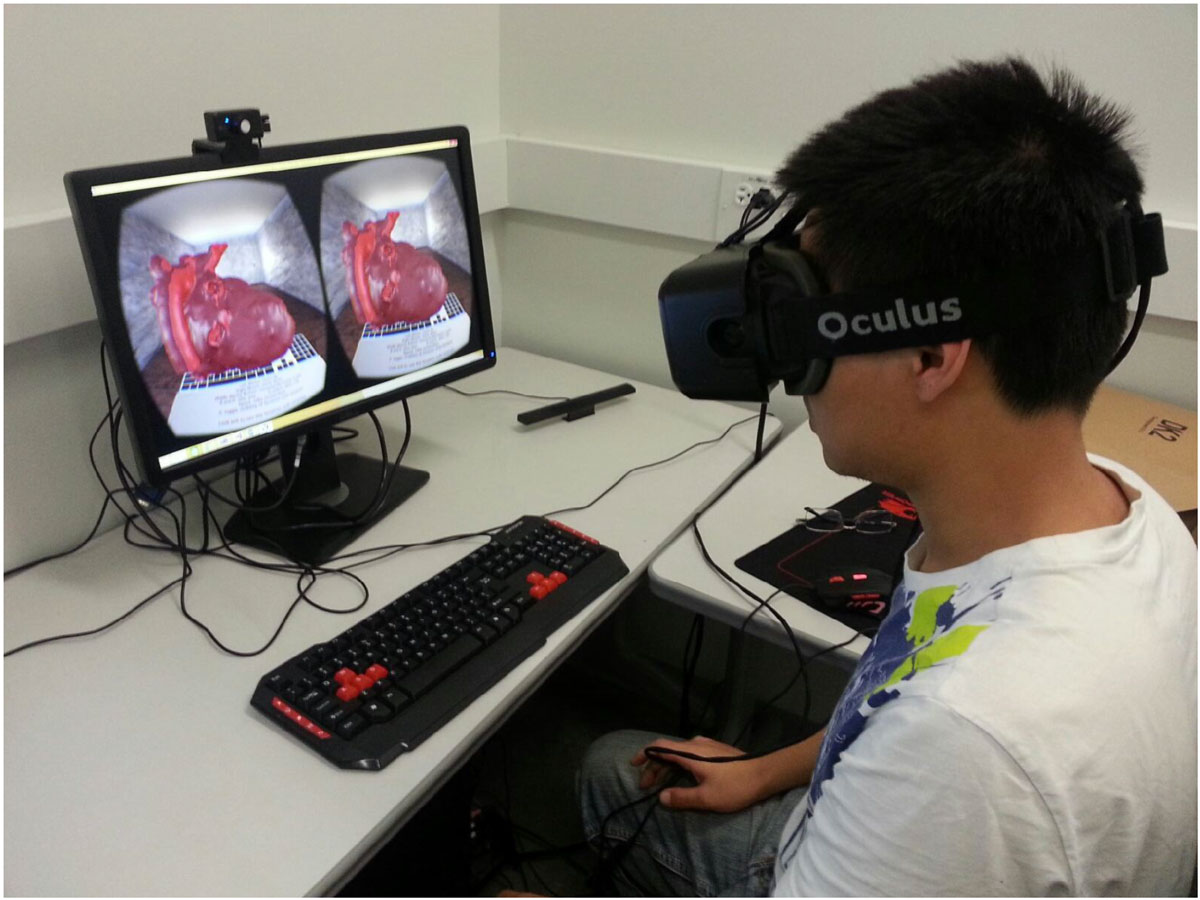
**Immersive visualization of a patient specific heart model using Occulus head mounted display**.

